# Cortical visual area CSv as a cingulate motor area: a sensorimotor interface for the control of locomotion

**DOI:** 10.1007/s00429-021-02325-5

**Published:** 2021-07-08

**Authors:** Andrew T. Smith

**Affiliations:** Department of Psychology, Royal Holloway, University of London, Egham, TW20 0EX UK

**Keywords:** Self-motion, Optic flow, Motor control, CMA, CCZ, SMA

## Abstract

The response properties, connectivity and function of the cingulate sulcus visual area (CSv) are reviewed. Cortical area CSv has been identified in both human and macaque brains. It has similar response properties and connectivity in the two species. It is situated bilaterally in the cingulate sulcus close to an established group of medial motor/premotor areas. It has strong connectivity with these areas, particularly the cingulate motor areas and the supplementary motor area, suggesting that it is involved in motor control. CSv is active during visual stimulation but only if that stimulation is indicative of self-motion. It is also active during vestibular stimulation and connectivity data suggest that it receives proprioceptive input. Connectivity with topographically organized somatosensory and motor regions strongly emphasizes the legs over the arms. Together these properties suggest that CSv provides a key interface between the sensory and motor systems in the control of locomotion. It is likely that its role involves online control and adjustment of ongoing locomotory movements, including obstacle avoidance and maintaining the intended trajectory. It is proposed that CSv is best seen as part of the cingulate motor complex. In the human case, a modification of the influential scheme of Picard and Strick (Picard and Strick, Cereb Cortex 6:342–353, 1996) is proposed to reflect this.

## Background

Cortical area CSv was first described by Wall and Smith (2008). At that time, numerous cortical areas that respond well to moving visual stimuli had been identified in both macaque and human brains. A growing focus on self-motion was evident; primates, including humans, are constantly on the move and effective movement requires continuous monitoring of the trajectory of self-motion. When we move, distinctive patterns of motion referred to as optic flow (Gibson 1950) occur in the retinal image and such flow provides a rich source of information from which our direction of heading during locomotion can be extracted (Warren and Hannon 1988). For many years, the analysis of optic flow was associated principally with the macaque cortical area known as MSTd (Saito et al. 1986; Tanaka and Saito 1989) although similar response properties had been documented in other regions of the macaque brain (see Fetsch et al. 2013; Smith et al. 2017 for reviews), notably VIP (Schaafsma and Duysens 1996). Micro-stimulation of MSTd can bias behavioural judgements of perceived visual heading in awake monkeys (Britten and van Wezel 1998) and reversible inactivation of MSTd impairs visual heading judgements (Gu et al. 2012), suggesting direct involvement of MSTd in heading perception. Based on functional magnetic resonance imaging (fMRI) data, a human homologue^1^ of MSTd was proposed (Dukelow et al. 2001), usually referred to as hMST, and selectivity for different optic flow components was demonstrated in hMST by adaptation (Wall et al. 2008). Transcranial magnetic stimulation (TMS) over hMST increases the variance of heading judgements (Schmitt et al. 2020) suggesting the involvement of hMST in the perception of self-motion trajectory.

## Human CSv

**Fig. 1:**
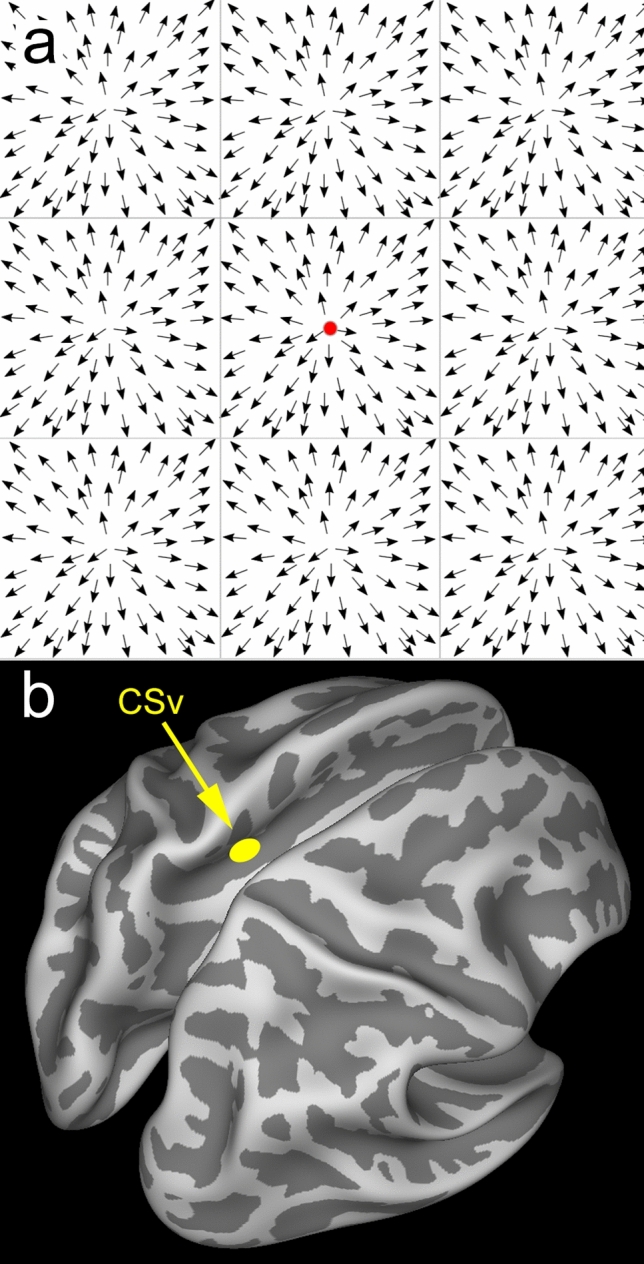
**a** Schematic visual stimulus with a central fixation point. Each arrow represents a dot that moves in the direction shown. The dots form a 3 × 3 array of square patches in each of which the dots move outward from the centre of the patch. Considered in isolation, each patch simulates the effect of forward motion through the environment but the overall stimulus does not. **b** Location of CSv shown in the left hemisphere of a partially ‘inflated’ human brain. The image is from a template brain available in CARET (Van Essen et al. 2001) that was created from MR images of many brains. Light grey represents convexities, dark grey concavities (sulci). CSv is situated in the posterior part of the cingulate sulcus with average left-hemisphere Talairach coordinates of [− 10 − 23 39] (Cardin and Smith 2010). It is also present at the corresponding location in the right hemisphere (not visible); left and right CSv are thought to have symmetrical functions

Wall and Smith (2008) reasoned that any cortical area that is specialised for extracting self-motion information from the retinal image should be active in the presence of naturalistic optic flow but should not respond well to any visual motion stimulus that is uninformative in relation to self-motion. They devised a stimulus to explore this distinction in an fMRI experiment. Responses to a large conventional random-dot pattern that simulated self-motion were compared with responses to a 3 × 3 array of smaller but otherwise similar flow patterns (see Fig. 1a). Each smaller flow patch would be consistent with self-motion if presented alone but the overall array of 9 patches is not. Rather than a simple expanding pattern simulating forward motion, a stimulus that cycles through spiral space, simulating constantly changing self-motion, was chosen. The reason was that in this way every possible dot direction was presented at every location at some point in the cycle, ruling out the possibility that any differences in overall response to the two stimuli might reflect differences in local motion. However, this choice had a fortuitous benefit that may explain why CSv had not been identified much earlier: changes in self-motion turn out to be crucial to obtaining a strong BOLD response in CSv (see below).

The responses to the 1-patch and 9-patch stimuli were very similar in the early visual areas (V1–V3), as might be expected. However, in the original experiment together with a more detailed follow-up study (Cardin and Smith 2010), five cortical regions were identified that responded significantly more strongly to the single, self-motion-compatible flow patch than to the array of 9 patches, indicating a preference for optic flow of a type that indicates self-motion. Three of these (hMST, hVIP and hV6) were already established as putative (though not unquestioned) human homologues of well-established motion-sensitive regions in the macaque brain. Another, thought at the time to be PIVC (parieto-insular vestibular cortex), can now be seen to be an adjacent area known as PIC (posterior insular cortex; Sunaert et al. 1999). The fifth, the focus of this review, was a small region in the fundus of the cingulate sulcus, in Brodmann’s area 23. The magnitude of the response difference between the 1-patch and 9-patch motion stimuli varied widely among these areas. In hMST, it was only about 10%. Thus, it seems likely that flow-sensitive MST cells convey only that the motion in their receptive fields (RFs) might reflect self-motion when considered alone and do not carry information about whether the total retinal flow, which extends beyond their own RF, suggests self-motion. Such a cell would be expected to respond well to 9 flow patches if its RF falls largely within one of the patches. Given that fMRI pools the activity of large numbers of neurons, the 10% reduction compared to the 1-patch stimulus might simply reflect the reduced contribution of cells with RFs that happen to straddle a boundary between two patches so that their optimum flow stimulus is never present. In hVIP and hV6, the response to 9 patches was reduced by around 60% compared to the response to one patch, suggesting much more highly developed sensitivity to whether the global stimulus reflects self-motion. PIC showed about 80% reduction. The largest reduction, about 90%, occurred bilaterally in a small region of the cingulate sulcus (Fig. 1b) that Wall and Smith named the cingulate sulcus visual area (CSv). This region was the most sensitive of any region in the brain to this experimental manipulation. On this basis, it was suggested that hVIP, hV6, PIC and CSv are all candidates for encoding visual cues to self-motion. CSv, being the closest to silent in the presence of multiple optic flow patterns that do not signal self-motion, appears to have the most highly developed ability to discount global cues that suggest there is in fact no self-motion.

Several previous studies had noted visual motion responses in the posterior cingulate cortex (PCC), without exploring its specificity. In parallel with Wall and Smith (2008), a second group (Antal et al. 2008) independently highlighted an area corresponding to CSv, referring to it as dPCC (dorsal PCC). In that study, it was shown with fMRI that CSv/dPCC responds to optic flow stimuli but not to random motion, consistent with the above interpretation. It has since been shown that CSv is actually suppressed by random motion (Pitzalis et al. 2013b; Wada et al. 2016). Fischer et al. (2012) showed that CSv has a strong preference for full-field motion and only a weak preference for contralateral stimuli, consistent with engagement with the overall visual image. Recently it has been shown (Pitzalis et al. 2020) that CSv responds during naturalistic simulations of self-motion and not during equivalent motion that simulates objects moving around a static observer.

Wall and Smith (2008) observed in a control experiment that responses in CSv were much larger when flow cycled through spiral space than when continuous expansion simulating constant forward motion was presented. To investigate this more formally, Furlan et al. (2014) compared responses to simulated motion across a ground plane that reflected either forward linear motion with a constant heading direction or the same forward motion with the addition of a sinusoidal lateral component such that instantaneous heading direction was constantly changing. In CSv, the BOLD response was about four times larger during changing heading than constant heading, a difference much greater than seen in any other visual area. The response in CSv was abolished when motion was scrambled, in line with earlier studies (Antal et al. 2008; Pitzalis et al. 2013b). Confirming this preference for changing heading, a recent study (Di Marco et al. 2021) found that CSv responds more strongly to motion on a curved trajectory than to forward motion with constant heading. Furlan et al. (2014) conducted an additional experiment employing motion that simulated turning either to the left or to the right while moving forward and found that the direction of heading change could easily be decoded in CSv with multivariate pattern analysis (MVPA). The same was true in hVIP, but not elsewhere. This suggests the presence of neurons in CSv that not only respond when heading changes but respond selectively according to the direction of change.

The suggestion that CSv is involved in monitoring self-motion is given additional weight by the fact that compelling vestibular activity has been demonstrated in CSv, with artificial vestibular stimuli applied in conjunction with fMRI (Smith et al. 2012; Billington and Smith 2015; Aedo-Jury et al. 2020). Sophisticated integration of visual and vestibular cues to heading has been demonstrated in MSTd, VIP and VPS of macaques (see Fetsch et al. 2013; Gu 2018 for reviews) and it seems likely that similar processes occur in humans. CSv is potentially an additional site of visual–vestibular interaction. Surprisingly, however, an attempt to decode congruent and opposite visual–vestibular combinations (Billington and Smith 2015) showed a complete failure to do so in CSv, despite good success in other visual areas (hMST, hVIP and PIC) in the same study. CSv receives both visual and vestibular information relevant to self-motion but evidence for integration of signals from the two modalities is lacking, although integration cannot be ruled out.

CSv may also receive eye movement signals. Berman et al. (1999) conducted an experiment in which the participants’ eyes moved (saccades or smooth pursuit) during fMRI scanning. Activity was seen, particularly during smooth pursuit, at a location referred to as CGp that corresponds well with CSv. Although vestibular information arises in head-centred coordinates, visual information does not. Direction of heading in retinal coordinates requires interpretation in head-centred terms to be useful for determining self-motion and this may be facilitated by knowledge of eye position. The presence of eye-movement-related activity may indicate that CSv is part of this process.

## Macaque CSv

A macaque counterpart of CSv has not been identified with single-cell neurophysiology. Neurophysiological exploration of the cingulate cortex in general has been limited, perhaps because of the technical difficulty of recording deep in medial areas of the brain. A few studies have reported visual activity in posterior cingulate regions that could possibly correspond to CSv (Olson et al. 1993; Dean et al. 2004). One study (Guldin et al. 1992) identified a vestibular cingulate region in squirrel monkey and a recent neurophysiological study (Liu et al. 2021) has reported strong vestibular responses in the macaque posterior cingulate. These results might suggest the existence of CSv in non-human primates but do not do so clearly.

**Fig. 2:**
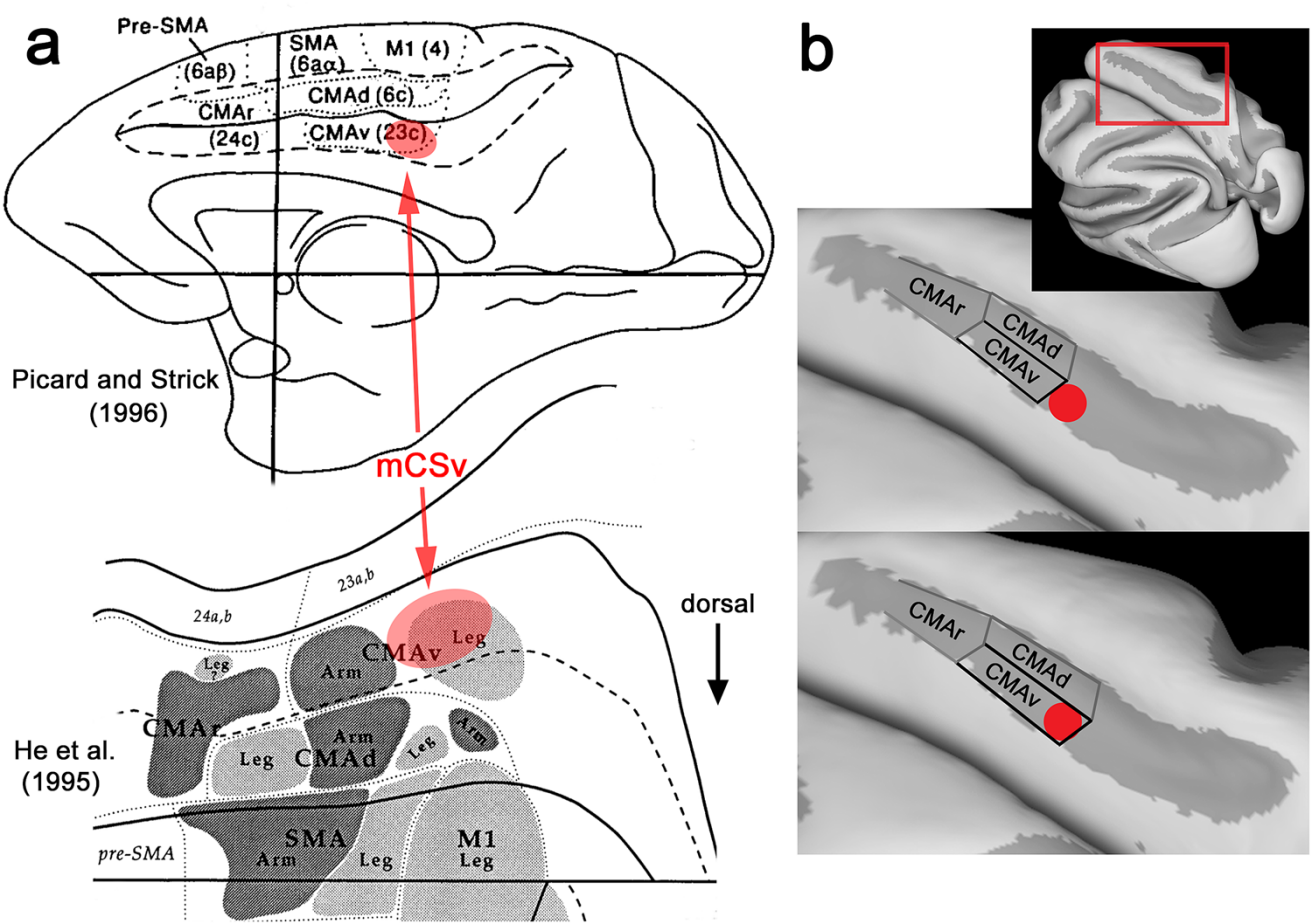
**a** Drawings of the medial surface of the macaque brain from Picard and Strick (1996) (reproduced with permission) and He et al. (1995) (Copyright 1995 Society for Neuroscience) with the approximate location of mCSv superimposed. Like human CSv, mCSv is found in both hemispheres. **b** Two alternative interpretations of the position of mCSv in relation to motor area CMAv. The cingulate sulcus is shown as if opened up to reveal its upper and lower banks. The position of mCSv as determined with fMRI by Cottereau et al. (2017) is indicated (solid disk) in the lower bank of the sulcus. Inset shows the location of the sulcus in the “inflated” macaque brain based on the F99 template in CARET (Van Essen et al. 2001)

Despite the absence of clear neurophysiological evidence for macaque CSv, a putative macaque counterpart of CSv has been identified with fMRI (Cottereau et al. 2017). In this study, the 1-patch and 9-patch stimuli of Wall & Smith (2008) were presented to alert fixating monkeys during fMRI and the responses contrasted. A test for differential activity (1 > 9) revealed a similar set of cortical regions to that seen in humans. One of these was a small region of the posterior cingulate sulcus, again in area 23, that the authors referred to as pmCSv (putative macaque CSv). Its location is shown in Fig. 2a. As in humans, the response in this region to a large changing flow field was substantially reduced (by about 75%) when the stimulus was replaced by an array of changing flow patches. Several other cortical regions showed the same trend, including MSTd which showed a 25% reduction, rather more than the 10% seen in hMST. However, pmCSv showed the greatest reduction of any cortical region, mirroring the human result. It was closely followed by area VPS (visual posterior Sylvian; Chen et al. 2011a) which is probably the macaque counterpart to PIC (Frank et al. 2014), PIC being the second most self-motion-specific human cortical area (Cardin and Smith 2010). So far, no macaque fMRI studies have used vestibular stimulation.

## Relationship of CSv to the cingulate motor areas

In this section, a proposal is made that incorporates CSv into the motor system, in both human and macaque. In the human case, this requires making an adjustment to the prevailing view of the cingulate motor areas. The argument is constructed in stages; readers preferring to know the bottom line at the outset may wish to look ahead to Figs. 2a (macaque) and 3d (human) and to the final paragraph of this section.

The macaque cingulate sulcus hosts three motor areas (Dum and Strick 1991; Picard and Strick 1996), known as CMAr, CMAd and CMAv (Cingulate Motor Area, rostral, dorsal and ventral). These occupy parts of Brodmann’s areas 6, 23 and 24.^2^ One of them, CMAv, is located in the ventral bank of the cingulate sulcus, in a sub-region of area 23 widely known as area 23c (Fig. 2a). Macaque CSv (mCSv as it will be called here) is also located in the ventral bank of the cingulate sulcus in area 23c (Cottereau et al. 2017). Thus, mCSv is at least very close to CMAv and it is pertinent to ask whether the two areas might be one and the same. Comparison of the fMRI definition of mCSv (Cottereau et al. 2017) with the location and extent of CMAv (Picard and Strick 1996) suggests that mCSv falls in the vicinity of the caudal (posterior) boundary of CMAv (see Fig. 2a). On this basis it is possible either that mCSv is a caudal sub-region of CMAv or that it is a separate region lying immediately posterior to CMAv; these two possible arrangements are illustrated in Fig. 2b. It will be argued here that the former is the case, although the issue is difficult to resolve with certainty.

The hindlimbs are represented caudally within CMAv, the forelimbs more rostrally (Morecraft and van Hoesen 1992; He et al. 1995). The central theme of the current review is that CSv is concerned with locomotion. If locomotion involves primarily the hindlimbs and mCSv is part of CMAv, then mCSv would be expected to fall in the caudal portion of CMAv. At the same time, if CSv is separate from CMAv it might make sense that it is located adjacent to the hindlimb representation in CMAv. Superficially, both arrangements are plausible. However, close inspection of anatomical data suggests that mCSv is probably within the boundaries of CMAv. Although the exact location of the caudal border of macaque CMAv is a little variable between published images, one estimate (He et al. 1995), based on spinal projections and illustrating the somatotopic organization, shows CMAv extending posteriorly to the point of inflexion in the sulcus (Fig. 2a, bottom). This leaves no room for a separate mCSv, which is more anterior than the inflexion, but instead places mCSv firmly in the leg representation of CMAv. Indeed, it is difficult to find published images in which CMAv does not extend far enough caudally to encompass the observed location of mCSv. On this basis, localization of mCSv within CMAv seems the likely arrangement.

**Fig. 3:**
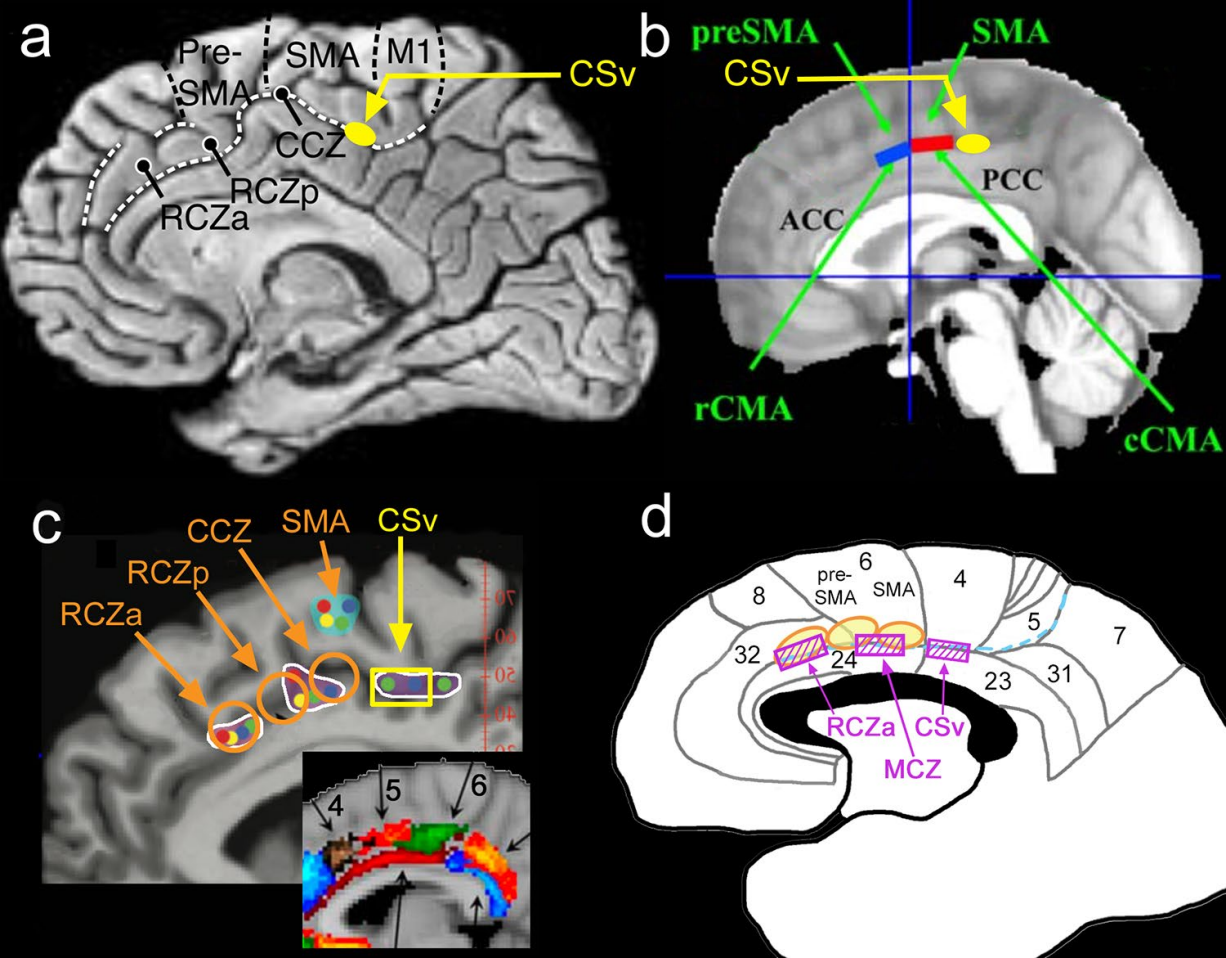
Location of human CSv in relation to the cingulate motor areas. **a** Medial surface of the right hemisphere, reproduced with permission from Picard and Strick (2001), showing the locations of their RCZa, RCZp and CCZ. The estimated location of CSv in this brain is superimposed (solid ellipse) and is more posterior. **b** Image modified from Habas (2010) (reproduced with permission) showing the locations of rCMA and cCMA (solid rectangles). Again, the estimated position of CSv is added. **c** Image modified from Amiez et al. (2014) (reproduced with permission) showing the average locations of their observed motor-related activity (small disks, representing different body parts) and their proposed clustering into three motor areas. For clarity, each cluster has been marked (irregular white outlines). Also superimposed are the expected location of CSv (hollow rectangle) and the approximate locations of RCZa, RCZp, CCZ (hollow circles) based on the image in (**a**). The inset is modified from Beckmann et al. (2009) (original image copyright Society for Neuroscience) and shows three of their cingulate zones (numbered 4–6) defined in terms of shared connectivity. These correspond well to the white outlines in the main panel. **d** Diagram of the medial surface showing the positions of the proposed cingulate motor areas (hatched rectangles) in relation to the relevant Brodmann areas (numbered, adjusted in light of the data of Vogt et al. (1995)). The positions of the three cingulate motor areas of Picard and Strick (1996) are also indicated (ellipses), based loosely on their Fig. 5D in which Brodmann boundaries were marked. The three areas proposed here correspond to the three clusters of motor activity identified by Amiez et al. (2014) but their correspondence to Picard and Strick’s areas is re-interpreted. One corresponds well to RCZa and its name is unchanged. One is CSv. The third, located between RCZa and CSv but corresponding neither to RCZp nor to CCZ of Picard and Strick, is termed MCZ (mid-cingulate zone). The three areas are shown separated by gaps because Amiez et al. describe three discrete clusters but it is possible that they might become contiguous if all possible body parts were mapped. The calcarine sulcus is marked by a dashed line. Although shown on the surface, the motor areas are largely buried in the sulcus

The same question can be posed in the case of human CSv. In the human brain, the arrangement of the cingulate motor areas is different from macaque. Building on their macaque work and previous human imaging studies, Picard and Strick (1996, 2001) postulated three human cingulate motor areas: RCZa, RCZp (Rostral Cingulate Zone, anterior and posterior) and CCZ (Caudal Cingulate Zone). Their locations are shown in Fig. 3a. Picard and Strick suggested that these human brain regions may correspond to their three macaque cingulate motor areas. However, their locations, in relation to each other, to the Brodmann areas and to CSv, are different from macaque. The most posterior of the three, and therefore the closest to CSv, is CCZ. In Brodmann’s terms, CCZ is located at the boundary of areas 24 and 6 (see Fig. 3c, d). Despite being the most posterior cingulate motor area, CCZ is therefore still more anterior than CSv, which is in area 23. Some authors (e.g. Habas 2010; see Fig. 3b) refer to just two divisions of human CMA, caudal (CMAc) and rostral (CMAr). These are described as being located beneath SMA (supplementary motor area) and pre-SMA, respectively, which again places even the more caudal area (CMAc) in area 24/6 rather than 23. It is clear that if this framework is correct, CSv cannot correspond to any of the human cingulate motor areas. One study has directly addressed the question of whether CSv is separate from the cingulate motor areas (Field et al. 2015). In an fMRI experiment, cingulate activity in a visual heading task (employing optic flow) was compared with activity from a motor (joystick) task. The authors confirmed the location of CSv in the fundus of the cingulate sulcus and found that their motor task elicited activity in a separate region, adjacent to CSv in the dorsal bank of the sulcus. They concluded that CSv is separate from cingulate motor activity. It should be noted, however, that joysticks engage the hands, locomotion engages primarily the legs and the caudal motor areas are known to be somatotopic, at least in monkeys (Morecraft and van Hoesen 1992; Luppino et al. 1993).

This leaves us in the position where CSv appears to be part of the cingulate motor areas in macaques but separate from them in humans. However, in the human case insight leading in a different direction arises by examining the results from an fMRI study of the human cingulate motor regions (Amiez and Petrides 2014). In this study, cortical activity during movements of the hand, foot, tongue and eyes was documented. The resulting activations were grouped into three somatotopically organized clusters which the authors suggested may correspond to the three cingulate motor regions of Picard and Strick (1996), RCZa, RCZp and CCZ. This suggestion is problematic in that CCZ of Amiez and Petrides is significantly more posterior than CCZ as described by Picard and Strick (see Fig. 3c), apparently falling in area 23 rather than area 24. RCZp of Amiez and Petrides is also an imperfect match to the original RCZp in terms of location. These discrepancies compromise the argument that the three areas of Amiez and Petrides are those proposed by Picard and Strick. Importantly, however, “CCZ” of Amiez and Petrides has a location very similar to that of CSv. Its location was reported to vary somewhat among brains and to be dependent on individual sulcal patterns (Amiez and Petrides 2014; Loh et al. 2018) so it is difficult to be sure that it actually coincides with CSv; this would require localization of CSv in the same participants. However, the uncertainty is largely resolved by the fMRI experiment of Serra et al. (2019) in which participants made rhythmical leg movements while supine and in addition several optic-flow-sensitive areas, including CSv, were identified in the same participants with visual localisers. CSv was found to be one of three visual areas (the others will be discussed in a later section) that were active during leg movements. This makes it likely that “CCZ” of Amiez and Petrides, although it is not CCZ of Picard and Strick, is in fact CSv. An earlier imaging study, involving movements of the arm, shoulder and knee (Fink et al. 1997), also identified three cingulate motor areas, the most posterior of which is in about the right location for CSv.

A possible resolution of the relationship between human CSv and the medial motor areas would therefore be that there are indeed three human cingulate motor areas (the white outlines in Fig. 3c, based on the grouping of Amiez and Petrides) but not all are located as proposed by Picard and Strick (1996) and one of them is, or encompasses, CSv. Consistent with this, the connectivity-based parcellation of the human cingulate region conducted by Beckmann et al. (2009) (see next section) yielded three regions (their Clusters 4, 5 and 6, shown in the inset of Fig. 3c) with locations that correspond well to the motor regions of Amiez and Petrides (2014) (white outlines in Fig. 3c). Beckmann’s Cluster 6 falls mainly in area 23c, where CSv is found, whereas CCZ of Picard and Strick appears to fall awkwardly at the boundary of Clusters 6 and 5. If the most caudal motor region is in area 23, rather than in area 24 where Picard and Strick placed it, this not only resolves the relationship of CSv to the motor areas in the human case but also has the benefit of bringing the human and monkey cases into alignment: in both species, CSv would be, or be within, the most caudal cingulate motor region. This goes against an influential schema but the schema, incorporating the belief that (in contrast to macaques) the human cingulate motor areas are all more anterior than area 23, is based on a suggestion (Picard and Strick 1996) that was not intended to be definitive. Moreover, the suggestion by these authors that human area 23 does not contain motor areas was based on the functional imaging data available at that time, when motor-related activity had not been observed in area 23.

Some care is needed here, however, because another interpretation is possible. The fMRI activity seen in human CSv during limb movement might reflect joint proprioception, rather than involvement in motor control. In fMRI studies, the presence of activity during voluntary movements is often seen as sufficient reason to declare a region to be a motor area but this is not necessarily a reliable criterion. Control experiments, for example showing that such activity is absent during equivalent passive movement of the limbs, would be needed before we can confidently regard activity as motor. Neither of the relevant studies (Amiez and Petrides 2014; Serra et al. 2019) included this or any other experimental manipulation to distinguish the sensory and motor interpretations. A better criterion is available in primate studies: if electrical stimulation of a brain area elicits movements, as it does in the cingulate motor areas (Luppino et al. 1991), then that area is probably part of a system that generates movement. The significance of the point is illustrated by the study of Berman et al. (1999) who defined an area they referred to as CGp based on fMRI activity seen when participants moved their eyes. The location of CGp appears to be the same as CSv. Is CSv, therefore, a motor area that is involved in controlling eye movements? Probably not. Eye-movement-related activity has also been seen in neurons of the nearby posterior cingulate gyrus (Olson et al. 1996) but such activity was seen shortly after the saccade, rather than preceding it, suggesting involvement in monitoring rather than generating eye movements. The same is apparent in the data of Dean et al. (2004) recorded at a similar location. In the case of limb-related activity in human CSv (Serra et al. 2019), whether such activity preceded or followed the onset of motor action is unknown and cannot be determined from fMRI data, which have poor temporal resolution.

Whatever the reason, CSv is active during leg movements (Serra et al. 2019). Importantly, equivalent arm movements did not activate CSv in that study. CSv is also active during passive observation of video footage depicting human locomotion (Abdollahi et al. 2014). These results are consistent with the proposition that CSv is concerned in some way with locomotion. Despite the need for caution in the interpretation of such fMRI data, the case is made here that CSv is involved in guiding motor activity. The case relies strongly on evidence of connectivity with known motor areas, reviewed in the following section.

In summary, it is suggested that in macaque there are three cingulate motor areas (CMAr, CMAd, CMAv), as widely accepted, and that mCSv forms the caudal portion of CMAv, where the hindlimb is represented. In humans, there are also three. The proposed arrangement of these is shown schematically in Fig. 3d. Only the most anterior, RCZa, is preserved from Picard and Strick. The second area does not correspond well to either RCZp or CCZ and is here termed MCZ (mid-cingulate zone). The third area is, or at least includes, CSv. CSv may correspond to a leg representation within the third area. This might be expected if macaque mCSv corresponds to the leg region of CMAv, but it has not been demonstrated. Unless and until the third region is shown to be more extensive or in some other way different from CSv, it would be premature to give it a different name.

## Connectivity of human and macaque CSv (MRI)

As for any other cortical region, a good way to gain an understanding of the place of CSv in the overall organization of the brain is to study its connectivity. The best method for assessing connectivity is the use of tracers that are injected directly into the area of interest and are then transported along axons to their target or source areas. This is not possible in humans. However, in recent years, connectivity in the human brain has been studied extensively with MRI. This approach is considerably more error-prone than tracer anatomy but has nonetheless yielded some impressive insights regarding organization of the human brain and is increasingly being applied in other species.

Several studies (Beckmann et al. 2009; Yu et al. 2011; Jin et al. 2018) have examined the connectivity of the human cingulate regions with structural methods, in which diffusion-weighted MR images are used to track white-matter pathways, and/or resting state functional MRI, in which correlations in spontaneous activity at rest between brain regions are assumed to reflect connectivity. One recent study (Oane et al. 2020) has even used intracranial stimulation to track cingulate connections. The emphasis in these studies was on parcellation of the cingulate into zones based on shared connectivity within zones and differing connectivity between zones. Two of these studies defined zones that are informative in relation to CSv. Beckmann et al. (2009) used structural connectivity data to divide the cingulate into nine clusters. One of them (their Cluster 6) was centred in area 23c, where CSv is located. The connectivity of this cluster was predominantly with parietal, motor and premotor cortex. Jin et al. (2018) used both connectivity methods and described six clusters based on functional connectivity and ten based on structural connectivity. One of their structural clusters (S7) had a similar location to CSv. Connectivity was strong with the precuneus, SMA and the middle superior frontal gyrus. Although these studies provide useful clues, neither study targeted CSv specifically and the clusters defined are larger than CSv. The cortical regions with which connectivity was specified are also large.

**Fig. 4:**
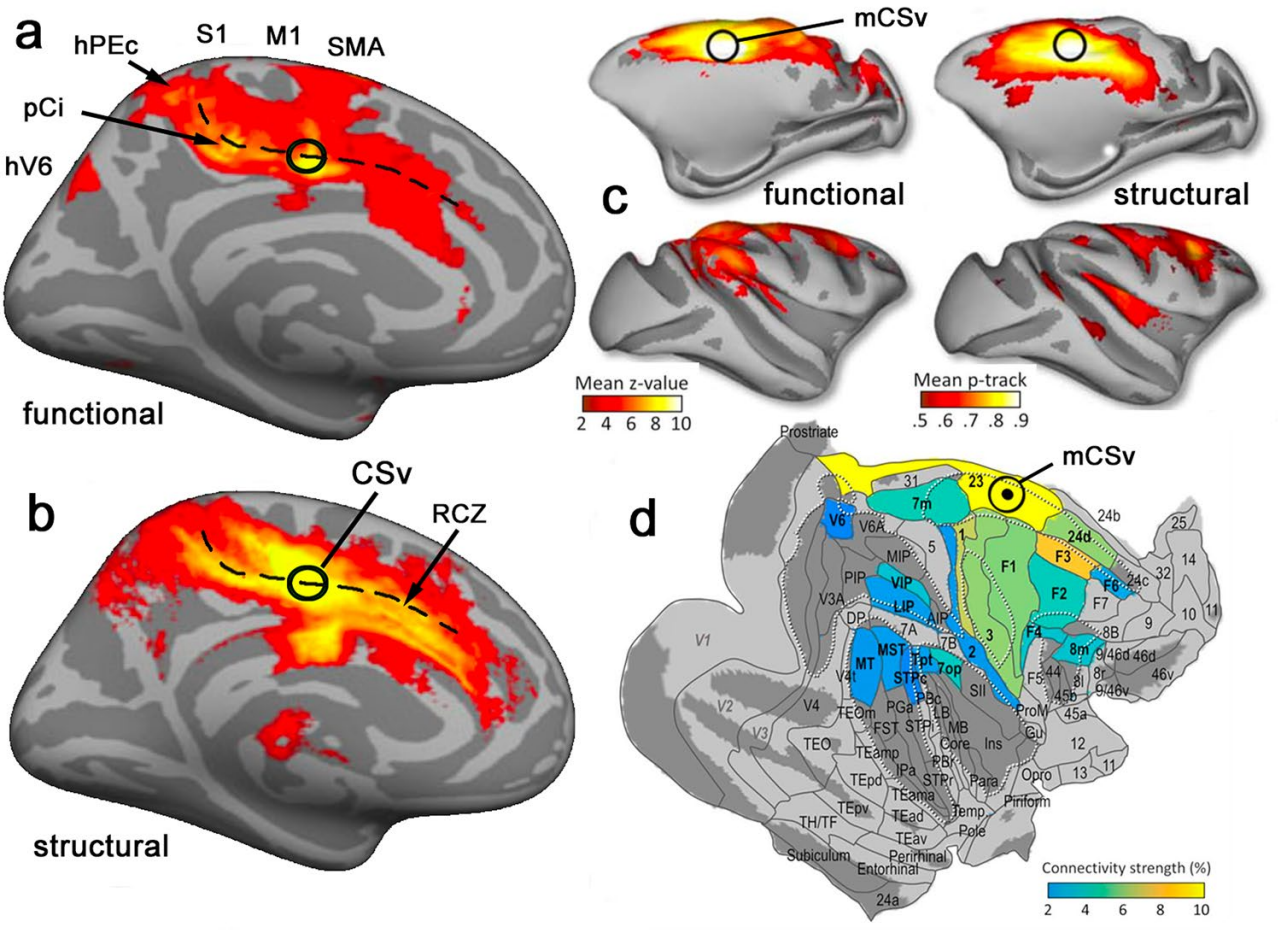
Connectivity of CSv and mCSv as determined with MRI methods. (**a**, **b**) Human, modified from Smith et al. (2018), showing converging results from **a** functional and **b** structural connectivity. Connectivity strengths are overlaid on the medial surface of an “inflated brain” created from MRI images. A black dashed line shows the fundus of the cingulate sulcus. The locations of various cortical regions (see text) are indicated, including CSv (black circle). (c and d) Macaque, modified from De Castro et al. (2021). **c** Results overlaid on an inflated brain template, separately for structural and functional connectivity estimates. **d** Average connectivity strength, derived by combining results from the two methods, for each cortical region delineated in the M132 atlas of Markov et al. (2014) that shows significant connectivity with mCSv

The connectivity of human CSv was specifically studied with MRI by Smith et al. (2018). Acknowledging the limited reliability of MRI-based connectivity, they employed both of the two commonly used methods (structural and functional) and looked for commonality between the two sets of results. CSv was defined with a functional localiser and used as a seed for connectivity analyses. A key aim was to identify the cortical regions that might provide sensory input to CSv. Two visual/vestibular areas (PIC and hVIP) and one further visual area (hV6) emerged as likely candidates. Connectivity between CSv and hVIP has since been confirmed independently (Raiser et al. 2020). However, these areas accounted for only a small fraction of the connectivity of CSv. The overall connectivity pattern is shown in Fig. 4a, b. The dominant feature was a streak of connectivity that followed the cingulate sulcus in both directions from CSv, anterior where the cingulate motor areas are located and posterior into the sensorimotor areas in and around the ascending portion of the cingulate sulcus (also known as the marginal sulcus or marginal ramus). The details of these cingulate areas will be elaborated later; some are marked in Fig. 4. Connectivity was also seen with the supplementary motor area (SMA). On this basis, CSv is perhaps better viewed as part of the sensorimotor system than as part of the perceptual system. Specifically, Smith et al. (2018) speculated that the function of CSv may be to feed sensory information about self-motion into the motor system to facilitate effective online control of locomotion, much as the posterior parietal cortex supplies visual information to the motor system for guiding reaching and grasping. This suggestion is the theme of the current review. Connectivity with somatosensory cortex was also observed, suggesting that CSv may receive proprioceptive, as well as visual and vestibular, signals that are relevant to locomotion. In support of the locomotor interpretation, somatosensory connectivity was found predominantly with the medial portion of primary somatosensory cortex (S1), in the paracentral lobule, where the legs and feet are represented. This selective connectivity with medial somatosensory cortex has been confirmed by Serra et al. (2019). The evidence for a role in motor control is extended by a recent study (Uesaki et al. 2019) in which the anatomical connections of CSv were classified according to which major white matter tracts they belonged to, based on re-analysis of the raw data of Smith et al. (2018). The largest component was associated with the dorsal component of the superior longitudinal fasciculus (SLF 1), which has been associated with motor planning and visuospatial integration (e.g. Howells et al. 2018).

A very similar dual-MRI approach to connectivity has recently been applied in macaques. De Castro et al. (2021) defined mCSv with a functional localiser and then estimated its cortical connectivity with both functional and structural MRI methods. The results, summarized in Fig. 4c, d showed broad agreement with those from human CSv. Cortical regions that showed reliable connectivity (significant connectivity evident in the results from both methods) included the cingulate motor areas and the supplementary motor area (SMA or F3). In addition, visual–vestibular areas VIP (thought to be related to human hVIP; Bremmer et al. 2001) and VPS (thought to correspond to human PIC; Frank et al. 2014) showed reliable connectivity. So did the somatosensory cortex and, as in humans, the medial (leg and foot) region showed stronger connectivity than the lateral (trunk and arms) portion. There were some differences between the macaque and human results. Notably, the primary motor cortex F1 (again, leg and foot regions most strongly) and frontal eye field region FEFsem appeared reliably to be connected with CSv in macaque whereas this was not the case in humans. Nonetheless the core pattern of connectivity is impressively similar in the two species: CSv is connected with medial motor areas, visual/vestibular areas, medial somatosensory cortex and the sensorimotor areas around the ascending cingulate sulcus. This connectivity pattern is consistent with providing an interface between the sensory and motor systems in the context of locomotion.

## Connectivity of macaque mCSv (tracer anatomy)

**Fig. 5:**
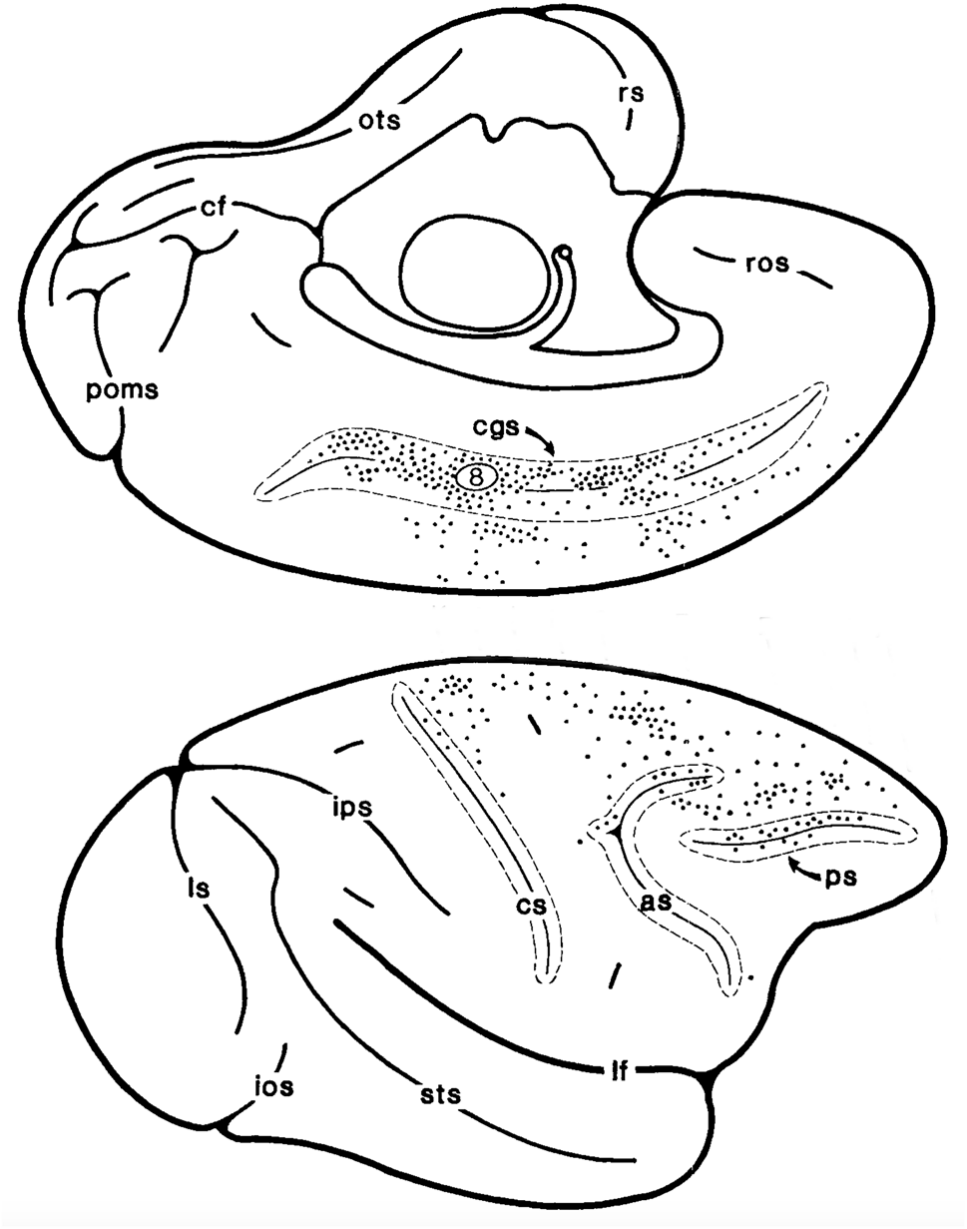
Medial (top) and lateral (bottom) view of a macaque cerebral hemisphere showing the labelling that occurred following injection of a retrograde tracer at the location marked “8” in the ventral bank of the cingulate sulcus, corresponding to area 23c. Each dot represents one stained cell. Reproduced with permission from Morecraft and Van Hoesen (1993)

A large literature documents studies in which axonal transport of injected tracer substances is used to identify connections between cortical regions. In this section, a detailed summary of tracer studies that may be relevant to the connectivity of mCSv is provided. Coverage of the literature is organized by reference to the MRI connectivity results summarized in the previous section, to facilitate comparison of results across methods. To anticipate, and to aid readers not requiring a detailed account, the overall picture is one of good agreement between MRI and tracer results, observed connectivity being predominantly but not exclusively with medial areas in and around the mid-cingulate and the ascending cingulate sulcus (marginal sulcus). Although it reflects only a single result, Fig. 5 will serve to convey this at a glance.

Although tracer anatomy has been used extensively in macaques, there are fewer studies of the medial than the lateral surfaces of the hemispheres, due at least in part to their relative inaccessibility, and there are only a few published studies in which tracer injections were made in area 23c (Bates and Goldman-Rakic 1993; Morecraft and van Hoesen 1993,1998; Morecraft et al. 2000,2004; Hatanaka et al. 2003). Typically these studies made just one or two injections in that region, as part of a larger study, and they tended to involve the rostral part of 23c whereas mCSv is more caudal. The case most likely to coincide with mCSv is perhaps Case 8 of Morecraft and van Hoesen (1993) in which, following an injection of retrograde tracer in caudal area 23c, labelled cells were found widely along much of the length of the cingulate, both anterior and posterior to 23c (see Fig. 5). More scattered labelling was found, among other places, in the supplementary motor area (SMA, also called M2). Hatanaka et al. (2003) also found labelled cells in the SMA following retrograde tracer injections in area 23c. Using anterograde as well as retrograde tracers, Bates and Goldman-Rakic (1993) showed that the connections between SMA and the cingulate sulcus are reciprocal.

In another, larger group of macaque tracer studies, labelled cells have been identified in area 23c following injections in other parts of the brain. By bringing these together, we can potentially assemble more of the connections of mCSv. Morecraft and van Hoesen (1992) injected retrograde tracers into either SMA (in medial area 6) or the primary motor cortex (area 4). In area 23c they found evidence of a strong projection to SMA, as well as a weaker one to motor cortex. By making injections in different parts of SMA representing different body regions, they demonstrated a somatotopic organization in area 23c (hindlimb and forelimb only, no face). Similarly, Luppino et al. (1993) made injections in SMA and also in pre-SMA. Following injections in the leg region of SMA, dense labelling was seen in area 23c. Labelling following injection in the arm region was more anterior, reflecting the same somatotopic organization, and more sparse. Injections in pre-SMA led to labelling in area 24 but not area 23. In both studies the somatotopy indicates that labelling was in motor area CMAv. The results are consistent with the connectivity of mCSv with SMA but not pre-SMA seen with fMRI, at least if we believe that mCSv is part of CMAv.

**Fig. 6:**
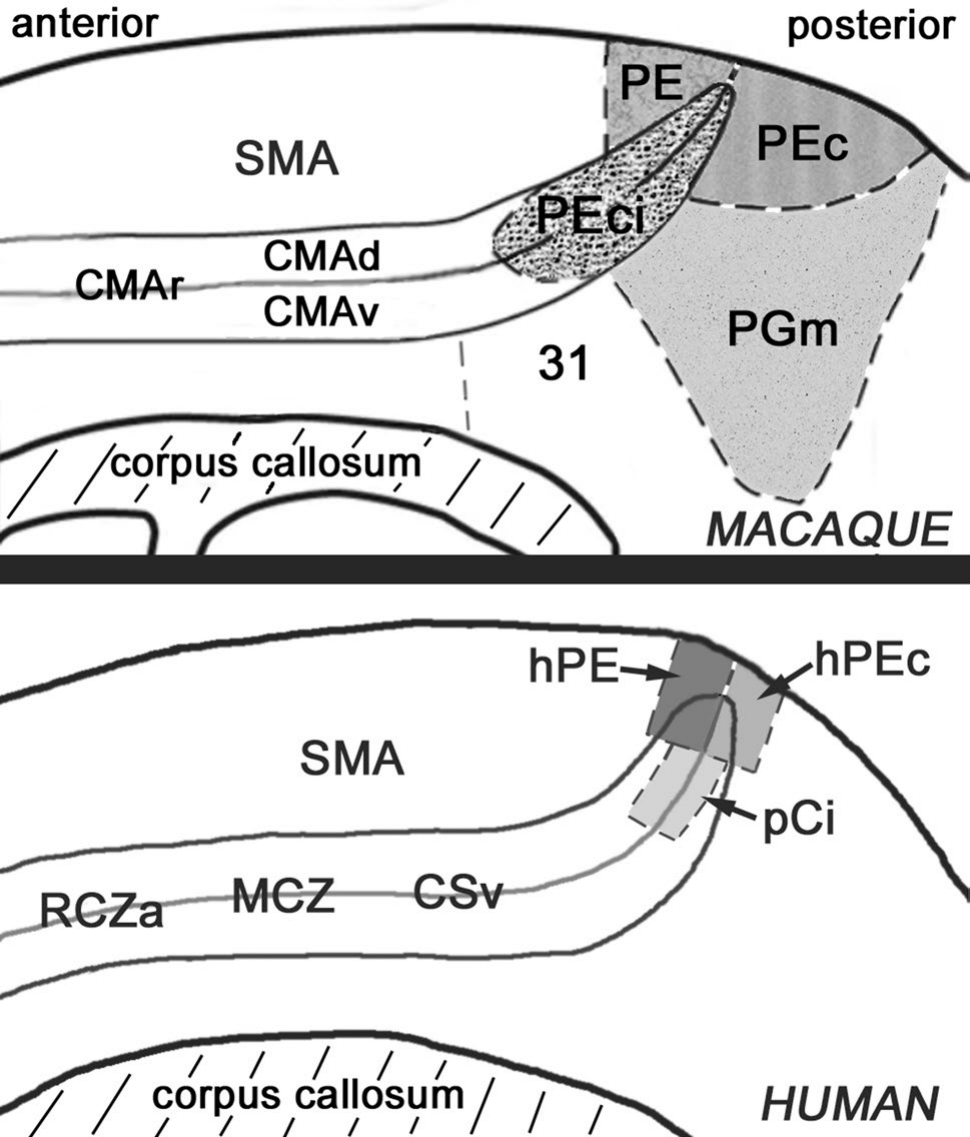
Diagram of a portion of the medial surface of the macaque (top) and human (bottom) cerebral hemispheres (not to scale) indicating the positions of the dorsomedial sensorimotor areas. In each case, the cingulate sulcus is shown opened up to reveal its dorsal and ventral banks. The locations of the proposed cingulate motor areas are also marked. See text for details of the various cortical areas. The macaque drawing is closely based on the drawings of Pandya and Seltzer (1982) (redrawn with permission). The human drawing shows the approximate locations of the dorsomedial areas (shaded areas) described by Pitzalis et al. (2019)

The above results emphasize the connections of area 23c with other medial motor areas, such as CMAr and SMA, that are located anteriorly and dorsally with respect to mCSv. However, the connectivity pattern seen with MRI in both macaque (De Castro et al. 2021) and human (Smith et al. 2018) also includes more posterior areas in and around the cingulate sulcus. Posterior to area 23, the cingulate sulcus turns dorsally towards the dorsomedial parietal cortex (see Fig. 6). Three separate areas have been distinguished in this vicinity (Pandya and Seltzer 1982): PE (immediately anterior to the tip of the sulcus, in what is conventionally seen as part of area 5), PEc (immediately posterior to it, in area 7) and PEci (in the sulcus, sometimes also called the supplementary somatosensory area SSA). PE is the largest of these areas. Most of it is on the lateral surface (so not visible in Fig. 6) but it extends a short distance onto the medial surface, where it meets PEc and PEci. It is characterized by proprioceptive signals arranged topographically with the leg represented medially, the arm and hand laterally. The arm and hand are over-represented and PE has been associated with manual dexterity rather than locomotion, but the medial portion adjacent to PEc/PEci is clearly concerned with the legs. Bakola et al. (2013) studied the connections of macaque PE by injecting retrograde tracers into different topographic zones. Among the results were labelled cells in area 23c. In the case of injections in the lower body representation of PE, labelling was quite dense and concentrated in one part of 23c, probably the caudal (leg) region of CMAv. Somatosensory afferents were also found. Area PEc also carries somatosensory signals but in addition has neurons that respond to optic flow (Raffi et al. 2010). The connectivity of PEc was studied by Bakola et al. (2010). Again, retrograde labelling was found in area 23c. Finally, Morecraft et al. (1998, 2004) provided evidence that PEci projects to area 23c.

Evidence also exists for connectivity between area 23c and medial parietal area PGm, which is located ventrally with respect to the above areas in medial area 7 (see Fig. 6) and has been associated with various cognitive functions including spatial cognition. In a tracer study of area 7 (Cavada and Goldman-Rakic 1989), one case involved an injection in area PGm and dense anterograde and retrograde labelling was seen in area 23c, demonstrating a reciprocal connection. Leichnetz (2001) also made tracer injections in area PGm; anterograde and retrograde labelling was seen throughout area 23 but was most dense in the ventral bank of the sulcus corresponding to area 23c. In one case documented by Parvizi et al. (2006) an injection in 7 m, likely in PGm, produced a dense, localised patch of retrograde labelling in area 23c. Passarelli et al. (2018) made an injection in PGm and did not find retrograde labelling in area 23c, but overall the evidence for bidirectional connections between PGm and 23c is strong.

Finally, area 31, located immediately anterior to medial area 7 and ventrally with respect to the posterior cingulate sulcus, might be expected to be connected with area 23 because of its proximity and because a recent study of the thalamic connectivity of the medial parietal cortex (Gamberini et al. 2020b) has grouped together PEci, area 23c and area 31 on the basis of a shared thalamic connectivity pattern. One case of Morecraft et al. (2004) involving a retrograde tracer in area 31 suggests that area 23c may project to it. So do two of the cases of Parvizi et al. (2006) but the labelling was in anterior 23c, thought by the authors to correspond to the arm area of CMAv. Passarelli et al. (2018) did not find retrograde labelling in area 23c following an injection in area 31. Although area 23c may project to area 31 there seems to be little evidence bearing on whether area 31 projects to area 23c and it is, therefore, unclear how important area 31 is in relation to mCSv. Connectivity estimated with MRI between mCSv and area 31 is not strong (De Castro et al. 2021).

The overall picture given by tracer injections in these dorsomedial regions is one of strong connectivity between area 23c (CMAv) and PE, PEc, PEci and PGm. In some cases, connectivity has been shown to be reciprocal and this may be true in all cases. This connectivity pattern is consistent with MRI connectivity data in macaques (De Castro et al. 2021) (Fig. 4c, d). A review of the sensory properties, connectivity and possible functions of these medial parietal regions has recently been provided by Gamberini et al. (2020a). These authors conclude that PE and PEc are concerned with the planning and control of limb movements. They argue that the connectivity of PEc, which does not appear to be somatotopically organized, suggests that it is more concerned with the legs than the arms and may be specifically involved in the control of locomotion. Additionally, it has been suggested (Breveglieri et al. 2008; Gamberini et al. 2018) that the sensory properties of PEc also indicate a role in locomotion. The connectivity of PEc with area 23c is therefore consistent with a role for mCSv in the control of locomotion, with PEc providing a key sensory connection. Human homologues of PE and PEc have been proposed (Pitzalis et al. 2019) and are within the dorsomedial zone that shows strong connectivity with CSv (Fig. 4a, b).

Another approach to the tracer literature is to examine the connectivity of the various visual and vestibular areas that were implicated as possible sources of sensory self-motion information in the recent MRI connectivity study of macaque mCSv (De Castro et al. 2021). The most compelling connectivity with optic-flow-sensitive visual areas in that study was with VIP. In a key study of the connectivity of VIP (Lewis and Van Essen 2000), three retrograde tracer injections in different parts of VIP yielded little or no labelling in area 23c. However, the retrograde tracer used would be expected to reveal only the inputs to VIP from other areas, not its outputs. The likely interpretation of the MRI connectivity between VIP and mCSv is that VIP is a source of sensory information for mCSv rather than a target for its output. One of the cases of Morecraft et al. (2004) involved an injection in area 23c that yielded some retrograde labelling in the intraparietal sulcus although it is unclear whether this included VIP.

De Castro et al. (2021) also reported strong connectivity between mCSv and the frontal eye fields (FEF) with both resting-state and structural MRI methods. However, this part of the cingulate sulcus does not feature strongly in studies of the connectivity of macaque FEF assessed either with retrograde (Schall et al. 1995; Wang et al. 2004) or anterograde (Stanton et al. 1995) tracers. A possible resolution is that these studies mostly concerned saccade-related parts of FEF (FEFsac) whereas De Castro et al. found more compelling connectivity with the smooth eye movement region (FEFsem). Macaque FEFsem, located deep in the arcuate sulcus, is more difficult to access than FEFsac. One study in Cebus monkeys, where it is more accessible, found scattered labelled cells in the posterior cingulate sulcus following an injection of a retrograde tracer in FEFsem (Tian and Lynch 1996).

Weaker, though significant, connectivity was apparent with MRI between mCSv and several other visual areas: MSTd, VPS, and V3A. A study of several cases involving tracer injections in MST (Boussaoud et al. 1990) showed no labelling in the cingulate sulcus. This study involved both retrograde and anterograde tracers, allowing us to conclude that as well as not projecting to MST, mCSv likely does not receive direct input from MST. The weak MST connectivity seen with MRI, confirmed recently by Raiser et al. (2020), may therefore reflect indirect connections. Tracer evidence relevant to whether area 23c is connected with V3A or VPS is elusive.

Weaker evidence still (structural but not functional MRI connectivity) suggested a possible link between mCSv and the superior temporal polysensory area STP (Bruce et al. 1981), where neurons sensitive to optic flow are found (Anderson and Siegel 1999). Seltzer and Pandya (1994) made several injections in different parts of STP and found some labelling in areas 23a/b (cingulate gyrus) but not in the sulcus. Again, the tracer was retrograde so this tells us only that there may be no projection to STP from CSv; the expected direction of sensory inputs would be the reverse. However, the available data on injections in area 23c (Morecraft et al. 2004) show no clear retrograde labelling in STP.

Overall, the results of tracer studies are consistent with the connectivity of mCSv seen in macaque with MRI (De Castro et al. 2021). The interpretation of tracer results in our context would be complicated if we did not accept that mCSv is part of CMAv (see earlier). Arguably, however, the fact that the connectivity of caudal area 23c as identified with tracers is similar to the connectivity of mCSv found with MRI adds to the evidence that mCSv is indeed part of CMAv.

## Function of CSv and mCSv

Various lines of evidence support the suggestion (Smith et al. 2018) that human CSv provides an interface between the sensory and motor systems in the control of locomotion. First, it is responsive to naturalistic optic flow that signals self-motion but it does not respond well to modified flow that is incompatible with self-motion. Second, it receives input from the vestibular system, one purpose of which is to detect and quantify self-motion. Third, it is located close to the medial pre-motor areas (SMA and CMA) and has strong connectivity with them. The same considerations apply in macaques and it is likely that mCSv has a comparable function to human CSv. In this section, the nature of the sensorimotor role of CSv/mCSv is considered and the question of whether CSv/mCSv provides a unique interface or is part of a larger interface system is discussed.

Detailed information is lacking concerning the exact nature of the information being fed by CSv into the motor system and its use. There is little reason to think that CSv is involved in high-level navigation processes. A largely separate navigation network handles orienting in the environment and navigating around it (see Kong et al. 2017 for a recent review). This network, in which the retrosplenial cortex may provide a central hub, has little connectivity with the cingulate sulcus. It is likely that CSv is not involved in deciding the destination or objective of locomotion but has the role of monitoring and adjusting the trajectory of movement, to keep it in line with the intended path while giving a rapid response to obstacles, irregularities in the ground surface and any other factors that may affect the actual trajectory. This would require some kind of feedback loop between motor intention and motor outcome and this might be provided by the strong link that exists between CSv and SMA, which is bidirectional at least in macaque. There is rather little evidence concerning neuronal response properties in macaque area 23c, where mCSv is located, and it relates only to arm movements as recordings have apparently been made only in the part of area 23c that represents the forelimb. Many cells here are active during arm movements (Cadoret and Smith 1995, 1997). Russo et al. (2002) showed that such responses have a shorter latency and longer duration than in SMA, consistent with a role in online control rather than initiation of movements. Crutcher et al. (2004) showed that such neurons represent the target of movement as well as the movement itself, which may reflect a way of anchoring movements to the intended path. If similar properties pertain in relation to the hindlimbs, it is easy to relate them to the requirements of effective locomotion. Indeed, the forelimbs themselves are used extensively by monkeys during locomotion. The fact that CSv is strongly responsive to optic flow only if the heading direction is changing and is near-silent during motion in a constant direction (Wall and Smith 2008; Furlan et al. 2014) is also consistent with a role in monitoring deviations from the intended trajectory. This property is also seen in hVIP, which might monitor deviations towards obstacles or threats in near space.

Several other cortical areas share some of the properties of CSv and we must therefore consider whether it is appropriate to regard CSv as one part of a locomotory interface system, rather than as a unique node connecting a group of sensory areas with a group of motor areas. A good starting point is to consider other areas that also have strong preferences for self-motion-compatible optic flow. In the human brain, the strongest preference (after CSv) is shown by PIC, followed by hVIP and hV6 (Cardin and Smith 2010). In the macaque, the areas with the strongest preference are VPS, followed by VIP and FEF (Cottereau et al. 2017). The common elements are thus VPS/PIC and VIP/hVIP, with possible species differences in relation to V6 and FEF.

There are good reasons to think that human PIC and macaque VPS are broadly equivalent. They have homologous locations, they both have visual and vestibular properties (Chen et al. 2011a; Frank et al. 2014) and they are both highly sensitive to whether optic flow reflects self-motion. PIC (in common with CSv and pCi) is active during leg movements (Serra et al. 2019), suggesting some kind of involvement in locomotor control. However, the connectivity of VPS/PIC with the motor system appears limited, although in the case of VPS it has not been examined in detail. An MR tractography study of the connections of PIC (Wirth et al. 2018) showed no connectivity with any of the established components of the human motor system. Nonetheless, PIC may be connected with SMA: this is evident in the functional connectivity data of both Serra et al. (2019) and Indovina et al. (2020). Connectivity between PIC and the dorsomedial region in and around the ascending cingulate sulcus (see discussion of pCi and hPEc below), has also been suggested (Indovina et al. 2020). PIC was shown by Smith et al. (2018), with both structural and functional MRI methods, to have connectivity with CSv, although this is also not evident in the MRI tractography of Wirth et al. (2018), and macaque VPS appears to be connected with mCSv (De Castro et al. 2021). Overall, the connectivity evidence suggests that PIC/VPS could be a source (either direct or indirect) of sensory information for CSv/mCSv (see next section) but it appears less well placed than CSv/mCSv to feed sensory information into the motor system. This said, the available connectivity evidence is far from comprehensive. Perhaps a more powerful argument against VPS/PIC providing a key sensorimotor interface for guiding locomotion is that nearly all VPS cells with visual and vestibular tuning have opposite visual/vestibular preferences (Chen et al. 2011a), suggesting that VPS does not carry integrated self-motion estimates but has some other function, such as using vestibular information to discount the effects of self-motion on the retinal image when estimating object-motion.

Homology between macaque VIP and human hVIP is less clear. Both respond well to optic flow (Bremmer et al. 2001, 2002a) with a preference for self-motion-compatible flow (Wall and Smith 2008; Cottereau et al. 2017). A possible difference concerns vestibular responsiveness. This is strong in macaque VIP (Bremmer et al. 2002b; Schlack et al. 2002), which has many neurons that combine visual and vestibular signals to optimize heading estimates (Chen et al. 2013), helpful for guiding motor activity as well as for perception. In human hVIP, vestibular sensitivity is not usually seen in imaging studies (see Smith et al. (2017) for discussion), although a recent study suggests that it emerges during simulated antero-posterior motion (Aedo-Jury et al. 2020). Both VIP and hVIP are connected with CSv/mCSv. Like CSv, hVIP is particularly sensitive to changes in heading direction (Furlan et al. 2014). Both VIP and hVIP, however, have features suggesting that they may not be suitable for feeding self-motion signals into the motor system in the way envisaged here for CSv. First, in the human case, there is fMRI evidence that hVIP is concerned with object-motion rather than self-motion. Pitzalis et al. (2020), who refer to hVIP as IPSmot to avoid assuming homology, found that it was more sensitive to object-motion than self-motion when the two were compared using naturalistic simulations. Field et al. (2020) also simulated real-world motion and found that hVIP responds more to objects moving past the observer than to the observer moving past objects. In macaque, the fact that inactivation of VIP does not impair heading perception (Chen et al. 2016) also raises doubt about its role in encoding self-motion. Second, in the macaque case there is evidence that VIP is concerned primarily with the space near the observer and particularly with objects approaching or touching the face (Colby et al. 1993; Bremmer et al. 2013). Microstimulation can elicit defensive movements that are consistent with object avoidance (Cooke et al. 2003). It is likely that VIP/hVIP does have an important role in guiding locomotion, possibly via its connectivity with CSv, but a specialised role: providing information about potential collisions with nearby objects.

V6 and FEF both have differences between macaque and human. Human V6 (hV6) prefers self-motion-compatible flow and is connected (directly or indirectly) with CSv. Macaque V6 neurons respond well to optic flow and are tuned for simulated heading direction (Fan et al. 2015) but there is no clear preference for self-motion-compatible flow (Cottereau et al. 2017) and V6 does not appear to have direct connections with mCSv (De Castro et al. 2021; Shipp et al. 1998; Galletti et al. 2001). Human V6 shows no vestibular activity in most fMRI studies although, as with hVIP, it may emerge if antero-posterior translation is simulated (Aedo-Jury et al. 2020). Macaque V6 is thought to have no vestibular sensitivity (Fan et al. 2015). V6 could be a source of flow information, especially in humans where CSv connectivity exists, but it is unlikely to be a sensorimotor interface for locomotion. Similarly, FEF (in both species) is associated with eye movements and visual attention, rather than self-motion, so it is unlikely to be at the centre of locomotor control. At least in macaques it is connected with mCSv (De Castro et al. 2021) and could be supplying information about eye position or craniocentric location. The activity seen in human CSv during eye movements, particularly smooth pursuit (Berman et al. 1999) is consistent with this.

Another group of areas that needs to be considered is those in and around the ascending portion of the cingulate sulcus (the marginal sulcus). In the macaque, these are PE, PEc and PEci, described earlier (Fig. 6, upper panel). PE has strong somatosensory inputs but not visual or vestibular sensitivity. Pitzalis et al. (2019) have identified a human homologue of PE (named hPE) with comparable characteristics at the corresponding location (see Fig. 6, lower panel). PE may be involved in planning limb movements (Gamberini et al. 2020a) but it can be discounted as a sensorimotor hub for locomotion. In contrast, PEc has visual and somatosensory responses (Raffi et al. 2002) and, unlike more posterior visual areas such as V6, visual responses are to some extent modulated by eye position (Raffi et al. 2010). It has previously been suggested (Breveglieri et al. 2008; Bakola et al. 2010) that PEc may be closely involved in the control of locomotion. In humans, Pitzalis et al. (2019) identified a homologue of PEc, just behind the dorsal tip of the cingulate sulcus, as in macaque, and called it hPEc. They showed hPEc to be responsive to optic flow, emphasising the lower visual field, and active during limb movements, including the legs. Maltempo et al. (2021) recently showed that activity in hPEc during foot movements was greater when the movement was towards the lower than the upper visual field. Consistent with a role in encoding self-motion, Pitzalis et al. (2020) found that hPEc responds better to self-motion than object-motion. Further, Pitzalis et al. (2019) performed a functional connectivity analysis that suggested hPEc may have connections with visual, vestibular, somatosensory and motor regions (including area 23c and SMA), as does PEc in macaque (Bakola et al. 2010). De Castro et al. (2021) found evidence of CSv connectivity in this region with functional, although not with structural, MRI methods. Pitzalis et al. (2019) outlined a strong case for the involvement of PEc and hPEc in locomotor control. However, given its location close to regions that are strongly sensory and the absence of a preference for flow that is self-motion-compatible, in either species (Cardin and Smith 2010; Cottereau et al. 2017), its primary role may be to feed visual and somatosensory data into the locomotion-control process rather than to provide a key sensorimotor interface.

Macaque PEci also has somatosensory activity but visual activity has not been reported and so, like PE, it is not a candidate as a visuomotor hub. No human homologue of PEci has been proposed, but near the corresponding location to PEci (see Fig. 6) lies a region, called Pc by Cardin and Smith (2010) and pCi by Serra et al. (2019), that has strong flow sensitivity with a preference for self-motion-compatible flow. In common with CSv and PIC, this region, which presumably cannot be a homologue of PEci and has no other proposed macaque equivalent, is more activated by leg than arm movements (Serra et al. 2019). It responds better to self-motion than object motion (Pitzalis et al. 2020), as do hPEc and CSv. The functional connectivity results of Serra et al. (2019) show pCi connectivity with CSv, CMA and SMA. Area pCi also shows connectivity with CSv in the results of Smith et al. (2018). With these properties, pCi may be the nearest rival to CSv as a candidate sensorimotor interface for locomotor control in the human brain. A hypothesis based on its location, and its selectivity for self-motion-compatible flow that is strong but does not match that of CSv, is that CSv provides the key interface and pCi is a node at an advanced level in a pathway connecting sensory cortex to CSv.

In summary, CSv (both human and macaque) is better placed than any other cortical area to provide the hypothesised sensorimotor interface for locomotion. However, some of its multisensory self-motion-related properties are also seen in other areas, suggesting that some of these properties may be inherited by CSv rather than being generated de novo. The sensory pathways feeding CSv and mCSv will be considered in the next section.

## Sources of sensory data for CSv

**Fig. 7:**
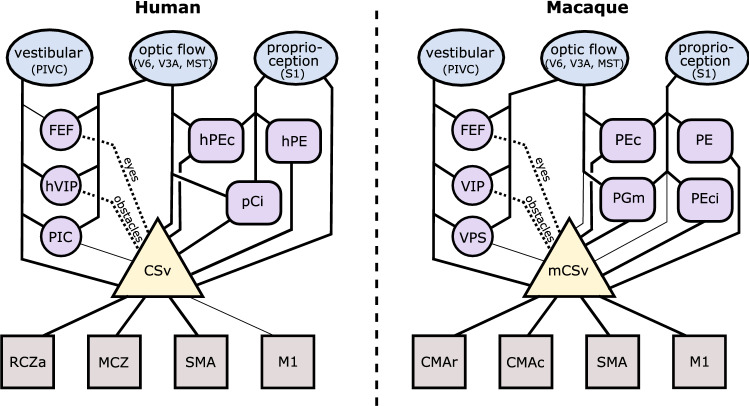
A possible scheme for the flow of information through human CSv (left) and macaque mCSv (right). Thick lines indicate strong connections, thin lines weak or uncertain connections. Dotted lines indicate pathways for transfer of specialized information. See text for details of the cortical areas identified. Various additional connections that do not involve CSv/mCSv exist between these areas and many of the connections shown are known to be reciprocal, so the actual information flow is less serial than depicted

It will be clear from the previous section that numerous cortical areas potentially provide sensory data to CSv/mCSv. These will now be listed and the evidence relating to each outlined. The surprising conclusion is reached that the obvious hypothesis, that integrated visual–vestibular self-motion signals are supplied to CSv by one or more of the well-known cortical areas that carry such signals, appears to be incorrect. Instead, visual and somatosensory signals may arrive via dorso-medial areas in and around the ascending cingulate sulcus while vestibular signals may arrive independently from vestibular areas such as PIVC. This is apparent in the scheme presented in Fig. 7. The detailed case now follows.

Somatosensory information, presumably emphasising proprioception, is able to reach mCSv from somatosensory cortex via some combination of PEc, PE, PEci and area 31, and to reach human CSv via a comparable system including hPEc and hPE. Direct input from somatosensory cortex is also suggested by MRI connectivity in both species (Smith et al. 2018; De Castro et al. 2021). Surprisingly, perhaps, it is more difficult to say how visual and vestibular signals reach CSv. Visual and vestibular heading cues need to be combined to estimate self-motion trajectory optimally. In the macaque, at least four brain regions (MSTd, VIP, FEFsem and VPS) do this very effectively (Gu et al. 2008,2016; Chen et al. 2011a, b) and we might expect to find that integrated visual–vestibular signals are fed to mCSv from such an area. In fact, it is difficult to make a strong case that any of them does this. VIP has already been discussed; it likely supplies signals to mCSv that mainly concern nearby objects to be avoided, although this is not certain. MSTd is strongly associated with optic flow and has a degree of selectivity for flow that is self-motion-compatible (Cottereau et al. 2017). However, MSTd does not appear to be directly connected with mCSv; it shows only weak connectivity with MRI methods (De Castro et al. 2021) and such a link is not apparent in classic tracer studies of MST connectivity (Lewis and Van Essen 2000). It is possible that MSTd provides combined visual–vestibular heading information to mCSv, perhaps indirectly, but there is no strong evidence. The frontal eye fields (FEF) have some connectivity with mCSv and were discussed in the previous section in the context of providing it with eye position information. Vestibular activity in FEF has usually been interpreted in terms of facilitating accurate eye movements during head motion (Fukushima et al. 2006). The presence of combined visual–vestibular heading signals may indicate a wider role but it seems unlikely that FEF is a key supplier of heading signals to mCSv. This leaves VPS, which is in some ways a likely candidate as it has very strong selectivity for self-motion-compatible flow as well as showing visual–vestibular integration. However its connectivity with mCSv is uncertain and, as discussed in the previous section, the fact that visual and vestibular preferences are opposite in most VPS neurons argues against the idea that it supplies an integrated visual–vestibular self-motion signal to mCSv.

In the case of human CSv, the situation is similar. At least three areas (hMST, hVIP and PIC) probably have separate populations of cells with congruent and opposite visual/vestibular tuning (Billington and Smith 2015) suggesting similar neuronal properties to their macaque counterparts. Of these areas, hVIP is likely involved only in the context of avoiding objects in near space and hMST has only very weak selectivity for flow that is self-motion-compatible (Wall and Smith 2008) and again has only weak, probably indirect, connectivity with CSv (Smith et al. 2018). PIC has connectivity, although not especially strong, with CSv according to one study (Smith et al. 2018) but not according to another (Wirth et al. (2018). Connectivity is weak, although statistically significant, in the data of Serra et al. (2019). On the plus side, PIC is active during leg movements (Serra et al. 2019) suggesting possible motor involvement, although the same study shows connectivity between PIC and somatosensory cortex so this activity could be proprioceptive. Moreover, if PIC is a homologue of VPS as believed (Frank et al. 2014) then it would be expected to have mostly opposite visual and vestibular preferences, arguing against its involvement. Human FEF may have vestibular input but the evidence for this is limited and its purpose is probably optomotor rather than locomotor.

Two further multisensory areas to be considered are STP (superior temporal polysensory area) and area 7a. Macaque STP has some connectivity with mCSv in the study of De Castro et al. (2021), though this was not mirrored in humans (Smith et al. 2018). STP (STPms in human) responds to visual, auditory and tactile stimuli in both macaques (Bruce et al. 1981) and humans (Beauchamp et al. 2008) but it has no reported vestibular activity and visual motion responses are associated more with object-motion than self-motion (Hietanen and Perrett 1997). It is possible that STP provides flow information to CSv but it is not particularly likely and it is most unlikely that it provides an integrated visual/vestibular signal. Macaque area 7a, situated in lateral parietal cortex and corresponding to area PG of Pandya and Seltzer (1982), has many neurons that are responsive to optic flow (Siegel and Read 1997). It has recently been shown (Avila et al. 2019) that vestibular responses are also common. Although this potentially creates a further candidate area for visual–vestibular integration, Avila et al. report that such integration does not occur in area 7a and indeed vestibular stimulation may suppress visual activity. They suggest that the role of 7a may be to resolve cue conflicts rather than to provide an integrated self-motion signal. There is in any case little evidence of connectivity between area 7a and mCSv. Whether a human homologue of area 7a exists is uncertain but there is no evidence of any visual–vestibular area in the vicinity that projects to CSv.

If CSv and mCSv do not receive integrated visual–vestibular signals, perhaps they receive separate visual and vestibular signals. Vestibular signals might come from PIVC, which receives strong, short-latency vestibular input from the brainstem via the thalamus. It is well documented that the posterior insula (where PIVC is located) is connected with the posterior cingulate in humans (Cauda et al. 2011; Taylor et al. 2009; Ghaziri et al. 2017). It has recently been suggested (Raiser et al. 2020) that PIVC (which corresponds to OP2; Eickhoff et al. 2006) is connected specifically with CSv and there are signs of this in the study of Smith et al. (2018). The posterior insula is also connected with the posterior cingulate in macaque (Vogt and Pandya 1987). PIVC does not respond to optic flow in either macaque (Chen et al. 2010) or human (Frank et al. 2016) and so could probably not provide optic flow signals but it is plausible that it could be the source of the vestibular sensitivity seen in CSv/mCSv.

Regarding visual input, Smith et al. (2018) suggested that hV6 may be a key source of optic flow data for human CSv, based on strong connectivity with CSv together with strong sensitivity to large-field optic flow patterns (Pitzalis et al. 2010) and moderate selectivity for self-motion-compatible flow (Cardin and Smith 2010). Macaque V6 also has neurons that respond well to optic flow but it does not have a preference for self-motion-compatible flow (Cottereau et al. 2017) and it does not have the strong connectivity with mCSv that is seen in humans. A further uncertainty about the involvement of macaque V6 is that it may be more concerned with object motion during self-motion than with self-motion per se (Galletti and Fattori 2003; Pitzalis et al. 2013a). At the same time, V6 is thought to feed into a dorsal motor system that is involved in controlling movements during reaching and grasping (Galletti et al. 2001) so it has motor credentials that may also make it a candidate for visual involvement in locomotion. Vestibular sensitivity is absent in macaque V6 (Fan et al. 2015), as it may be in hV6 (Smith et al. 2012, but see Aedo-Jury et al. 2020). V6 is quite likely to provide optic flow signals to CSv in humans but a direct connection, at least, is less likely in macaques.

Similar considerations apply to V3A (hV3A in humans), which has no known vestibular sensitivity in either species. In the study of Smith et al. (2018), this region had quite strong connectivity with CSv in humans when estimated with structural MRI methods but this did not hold up with functional methods. In macaque (De Castro et al. 2021), V3A showed significant mCSv connectivity with both methods although this was relatively weak. V3A has optic flow sensitivity in humans and has recently been shown to have stronger flow sensitivity in macaque than previously realised (Nakhla et al. 2021). It is, therefore, a possible source of optic flow information but evidence for a primary role is lacking. Its status is perhaps similar to hMST/MSTd: it has some of the right properties, some degree of connectivity with CSv/mCSv and may be involved but, if so, connectivity may be indirect.

Finally, PEc/hPEc and human pCi fall within the prominent streak of CSv/mCSv connectivity that follows the ascending cingulate sulcus (Fig. 4), and they also have optic flow sensitivity. Vestibular activity has not been reported in these areas. Studies of the connectivity of macaque PEc (Bakola et al. 2010) and human hPEc (Pitzalis et al. 2019) emphasise V6A as a source of visuo-motor signals, and V6A in turn receives visual signals from V6. Human pCi was discussed in the previous section as having much in common with CSv. When looking for the source of optic flow information in CSv/mCSv, we can envisage a pathway that begins with motion sensitivity in V3A and V6 and also includes PEc and pCi, becoming increasingly involved with motor planning before finally reaching CSv. The existence of species differences (particularly in V6, pCi, PEci) as well as possible homologies (such as in PE and PEc) dictates different details in the two species. Figure 7 shows a scheme for the possible flow of information through CSv in the two cases.

In summary, significant uncertainties remain concerning the sources of vestibular and (particularly) visual input to CSv and mCSv. For every visual area proposed, there are difficulties and counter-arguments. However, a key insight is that contrary to expectation, visual and vestibular signals may arrive by different routes rather than from an area that has integrated the two. As well as evidence from connectivity, a key reason to think this is the study of Billington and Smith (2015) who found that when visual and vestibular roll rotations were either summed (congruent) or subtracted (opposite), the difference could not be decoded in CSv (it could in PIC, hVIP and hMST). This suggests not only that CSv does not receive an integrated visual/vestibular signal from elsewhere but also that it does not create its own integrated signal. Rather, vestibular and visual signals that are not combined may be used to generate separate influences on motor co-ordination.

Perhaps we should not be surprised by the lack of evidence that integrated visual–vestibular signals are fed to CSv from areas such as MST. Cells that integrate visual and vestibular heading directions respond well to motion with a constant heading direction and their tuning was studied in those terms (Gu et al. 2008; Chen et al. 2011a). In contrast, human CSv responds well to optic flow only when heading direction is changing (see earlier). To derive signals specifying change of heading from a population of heading-tuned cells, whether in the visual or vestibular domain, would require significant additional processing. The same result might be achieved by extracting heading change more directly from lower-level visual and vestibular signals. Vestibular signals, with their inherent emphasis on change (acceleration), may be better suited to the task than visual signals. In both macaque and human CSv, the magnitude of visual responses recorded with fMRI is small compared to most other visually responsive areas, even with an optimal stimulus. During a recent neurophysiological investigation in which vestibular responses were documented in the macaque cingulate sulcus (Liu et al. 2021), visual responses were also sought using optic flow with a constant heading direction and such responses were weak (Gu, personal communication). The lack of strong evidence for a projection from MSTd, VIP or VPS to mCSv may be matched by a lack of evidence for cells in mCSv that have the visual–vestibular properties found in those areas. Tempting though it is to assume that integrated visual–vestibular heading signals like those in MSTd must be used for guiding locomotor movements, this may not be the reality.

## Conclusions and uncertainties

Human CSv and macaque mCSv have homologous locations and similar properties, although there are some differences, and they probably have related functions. We have seen that CSv has strong connectivity with the medial motor areas in both species, particularly the cingulate motor areas and SMA, suggesting that it is involved in motor control. Indeed, it may best be viewed as part of the cingulate motor complex. Whether or not that is accepted, the properties of CSv suggest that it provides a key interface between the sensory and motor systems in the context of the control of locomotion. It is likely that its role is in online control and adjustment of continuous locomotory movements, including obstacle avoidance. Considerable uncertainty remains and future progress will require advances on several fronts. Refinement of knowledge of the connectivity of CSv, coupled with refinement of knowledge of the functions of the areas with which it is connected, will assist in understanding the role of CSv more fully. Neurophysiological recordings obtained in mCSv, especially during locomotion or at least leg movements, could potentially yield a wealth of relevant information. In the human case, functional imaging during locomotion may assist although currently available techniques for imaging during active body movement, such as EEG and NIRS, have significant limitations of resolution and localization accuracy. The growing understanding of online motor control in relation to reaching and grasping may provide inspiration, as common principles may apply to locomotion.

## Data Availability

Not applicable.

## Footnotes

1 The words “homologue” and “homologous” are used here to refer to cortical areas in two species that have closely related functions and are assumed to derive from the same anatomical region in a common ancestor, whether or not they still have corresponding anatomical locations.

2 Different classificatory systems are in use for sub-divisions of both human and macaque cerebral cortex and these sometimes reflect differences not only in terminology but also in the positions of boundaries and the number of sub-divisions. In this review, Brodmann’s system is used to provide anchor points while refinements and sub-divisions are described in the terms chosen by the authors cited. Alternative names for the same area are added where this will aid clarity.
